# Role of sialidase in glycoprotein utilization by *Tannerella forsythia*

**DOI:** 10.1099/mic.0.052498-0

**Published:** 2011-11

**Authors:** Sumita Roy, Kiyonobu Honma, C. W. Ian Douglas, Ashu Sharma, Graham P. Stafford

**Affiliations:** 1 Oral and Maxillofacial Pathology, School of Clinical Dentistry, Claremont Crescent, University of Sheffield, Sheffield S10 2TA, UK; 2 Department of Oral Biology, School of Dental Medicine, University at Buffalo, The State University of New York, Buffalo, NY 14214, USA

## Abstract

The major bacterial pathogens associated with periodontitis include *Tannerella forsythia*. We previously discovered that sialic acid stimulates biofilm growth of *T. forsythia*, and that sialidase activity is key to utilization of sialoconjugate sugars and is involved in host–pathogen interactions *in vitro*. The aim of this work was to assess the influence of the NanH sialidase on initial biofilm adhesion and growth in experiments where the only source of sialic acid was sialoglycoproteins or human oral secretions. After showing that *T. forsythia* can utilize sialoglycoproteins for biofilm growth, we showed that growth and initial adhesion with sialylated mucin and fetuin were inhibited two- to threefold by the sialidase inhibitor oseltamivir. A similar reduction (three- to fourfold) was observed with a *nanH* mutant compared with the wild-type. Importantly, these data were replicated using clinically relevant serum and saliva samples as substrates. In addition, the ability of the *nanH* mutant to form biofilms on glycoprotein-coated surfaces could be restored by the addition of purified NanH, which we show is able to cleave sialic acid from the model glycoprotein fetuin and, much less efficiently, 9-*O*-acetylated bovine submaxillary mucin. These data show for the first time that glycoprotein-associated sialic acid is likely to be a key *in vivo* nutrient source for *T. forsythia* when growing in a biofilm, and suggest that sialidase inhibitors might be useful adjuncts in periodontal therapy.

## Introduction

The oral cavity houses a complex microbial community capable of progressive colonization of tooth surfaces, which can lead to the major dental diseases of caries and periodontitis (Socransky *et al.*, 1998). Periodontitis is a major cause of tooth loss, affecting over 300 million people worldwide (El-Kheshen, 2009), and bacteria associated with periodontitis are also linked with systemic problems such as endocarditis, atherosclerosis and predisposition to pre-term low birth weight (Tanner & Izard, 2006). The major bacterial pathogens associated with aggressive periodontitis are known as the ‘red complex’, which comprises *Tannerella forsythia*, *Porphyromonas gingivalis* and *Treponema denticola* (Socransky *et al.*, 1998). While considerable attention has been paid to the other members of this group, less is known of the virulence mechanisms or physiology of *T. forsythia*.

Our recent work has highlighted a major role for the host sugar sialic acid in the biofilm physiology and host–pathogen interactions of this key periodontal pathogen (Roy *et al.*, 2010). Sialic acids are commonly present as terminal sugars on the surface of a wide variety of host glycoproteins and eukaryotic cells (Angata & Varki, 2002; Varki, 1997). The sialic acid residues modulate a variety of biological functions, which include mediating cell–cell interactions, stabilizing glycoprotein structure and masking ligand-binding receptors (Angata & Varki, 2002; Varki, 1997; Bradshaw *et al.*, 1998). In addition to the identification of a novel transport system (NanOUT), we have highlighted the role of a secreted sialidase enzyme in acquisition of sialic acid from the sialoconjugate sugar sialyl-lactose, which contains sialic acid linked to galactose via an α-2,3 or α-2,6 linkage, and furthermore to glucose via a β-1,4 linkage (Roy *et al.*, 2010). Sialidases are a family of glycohydrolases which cleave the terminal sialic acid residue from sialoglycoproteins, and have been shown to play a role in virulence for a range of pathogens, including *Streptococcus pneumoniae* and *Pseudomonas aeruginosa* (Corfield, 1992; Reinholdt *et al.*, 1990). Their actions include inducing chemokine release from epithelial cells (Kuroiwa *et al.*, 2009), unmasking sialic acid-masked epitopes for adhesion (Corfield, 1992; Tong *et al.*, 2000), promoting biofilm formation (Soong *et al.*, 2006), and degrading host glycoproteins to obtain nutrients (Bradshaw *et al.*, 1994; Byers *et al.*, 1999; King *et al.*, 2006; Severi *et al.*, 2007). We have also shown that the *T. forsythia* NanH sialidase is important in interactions with human gingival epithelial cells (Honma *et al.*, 2011) in a mechanism that may expose underlying glycosyl epitopes for adhesion and invasion. However, while interaction with host cells is clearly an important process in periodontal pathogenesis, the ability of periodontal bacteria to form persistent and viable biofilms *in vivo* is equally pertinent.

It is well documented that the sialidase enzymes of some pathogenic bacteria contribute to virulence, especially of those that reside on and/or invade mucosal surfaces. This is likely due to the abundance of sialic acid on the host glycoproteins in these tissues (Corfield, 1992). Human salivary glycoproteins are no exception, and contain various complex sugar substrates such as mucin and fetuin (Pigman & Gottschalk, 1966). Mucin contains sialic acid linked via its terminal sugar by a 2-6′ glycosidic bond to *N*-acetylglucosamine (Mizan *et al.*, 2000), whereas fetuin contains both 2-3′ and 2-6′ sialic acid linkages and is more abundant in serum, but is also found in saliva and gingival crevicular fluid (Cointe *et al.*, 1998). We hypothesized that *T. forsythia*, like other human-dwelling bacteria, might be able to use the sugar substrates available in the oral cavity to obtain food and energy during the formation of dental biofilm.

We therefore set out in this study to examine the role of the NanH sialidase in biofilm formation using a range of relevant model sialoprotein-coated surfaces, and thus to begin to examine the putative *in vivo* substrates of this enzyme.

## Methods

### 

#### *T. forsythia*.

*T. forsythia* (ATCC 43037) was routinely grown either in liquid culture [TSB-NAM: 10 % trypticase soya broth (TSB; Oxoid) supplemented with 2 % yeast extract (YE; Sigma), 1 mg haemin ml^−1^, 1 mg menadione ml^−1^ (Sigma), 10 µg *N*-acetylmuraminic acid ml^−1^ (NAM; Sigma) and 50 µg gentamicin ml^−1^ (Sigma)] or on Fastidious Anaerobe agar plates (FA; Lab M) supplemented with 5 % horse blood containing 10 µg NAM ml^−1^ and 50 µg gentamicin ml^−1^. Broth cultures and FA-NAM plates were typically incubated at 37 °C in an anaerobic atmosphere (10 % CO_2_, 10 % H_2_ and 80 % N_2_) for 5 days. The sialidase mutant (Δ*nanH*) strain was grown in a similar manner but with 5 µg erythromycin ml^−1^ (Honma *et al.*, 2011).

#### Saliva collection.

Resting whole human saliva was collected from healthy individuals aged 25–35 years, centrifuged at 10 000 ***g*** for 5 min and filter-sterilized. The supernatant was used immediately for experiments.

#### Biofilm growth.

*T. forsythia* biofilms were grown essentially as described previously (Roy *et al.*, 2010). Briefly, 5 day-old *T. forsythia* colonies were harvested and washed twice in fresh TSB. For biofilm growth, bacteria were inoculated to a final OD_600_ of 0.05 into the supplemented TSB liquid medium in uncoated polystyrene tissue-culture plates (Greiner) and incubated anaerobically at 37 °C for 5 days. These biofilms were grown (without NAM) either with sialic acid (6 mM) or in the presence of the commercially available glycoproteins bovine submaxillary gland (type I-S), mucin (6 mM, contains 3.8 µg sialic acid, molecular mass 484 kDa), fetuin (6 mM, contains 0.12 µg sialic acid, molecular mass 68 kDa) and asialofetuin (6 mM, molecular mass 62 kDa) (all Sigma Aldrich). In addition, human serum (Sigma Aldrich) or fresh whole saliva was used at a dilution of 1 : 50 in PBS (~2 µg ml^−1^) in place of NAM or sialic acid. The glycoproteins were coated on the 96-well tissue-culture plates overnight at 4 °C, and then the wells were washed with PBS to wash off excess protein.

The cell number in mature biofilms was assessed as described previously (Roy *et al.*, 2010); briefly, after 5 days of growth and removal of the culture medium, samples were washed twice in sterile PBS, followed by vigorous resuspension of biofilm cells in PBS using a pipette, before counting microscopically using a Helber counting chamber (Hawksley) under a phase-contrast lens (magnification ×400). The effectiveness of the harvesting method was verified by crystal violet staining of the harvested wells to assess the number of residual cells attached to the plate, which was usually <0.01 % (Pham *et al.*, 2010).

#### Initial attachment assay.

For measurement of *T. forsythia* adhesion, essentially the same conditions as for mature biofilm growth were used, except that the assays were incubated for only 3 h at 37 °C anaerobically. After incubation, the cells were harvested and counted as described above.

#### Effect of sialidase (neuraminidase) inhibitor on biofilm growth.

The effect of the influenza virus sialidase inhibitor oseltamivir on *T. forsythia* biofilm growth was assessed by its inclusion at 10 mM in the medium, followed by the enumeration of biofilm cells after 5 days. This concentration of inhibitor was previously established in our laboratory as suppressing *T. forsythia* whole-cell sialidase activity (Roy *et al.*, 2010). As an alternative, siastatin B was used at a concentration of 5 mM (which inhibits at equivalent levels to the previously utilized oseltamivir), since during the course of this study supplies of oseltamivir were unavailable due to the influenza pandemic.

#### Purification of recombinant pGEX-NanH.

pGEX-*nanH* (this study) was transformed into *Escherichia coli* BL21 and a clone was induced using 0.1 mM IPTG. After addition of IPTG, the culture was incubated for 3 h to express the fusion protein. The cells were harvested by centrifugation, suspended in 200 mM phosphate buffer (pH 7), and subjected to cell disruption by French pressure cell (1000 p.s.i.; 6.9 MPa). The cell lysates were centrifuged at 10 000 ***g*** for 10 min at 4 °C to separate the soluble fraction from the cell debris. The recombinant fusion protein (rNanH) was purified from *E. coli* lysates as described previously (Honma *et al.*, 2011). The purified enzyme was checked for its activity by sialidase assay, with a mean batch activity of approximately 10–20 U.

#### Sialic acid release assay.

Glycoproteins (6 mM) were incubated with 0.78 mg rNanH ml^−1^ for 3 h at 37 °C in 50 mM sodium citrate buffer, pH 4.5. Free sialic acid was then measured using the method of Skoza & Mohos (1976). To ensure specificity the sialidase inhibitor siastatin B was included as indicated above.

#### Direct removal of lectin-binding domains on fetuin.

Fetuin (10 µg ml^−1^) was incubated with various concentrations of purified rNanH protein (0, 0.78, 1.56 and 3.12 mg ml^−1^) for 3 h at 37 °C in a total volume of 30 µl 50 mM sodium citrate, pH 4.5. The samples were then separated by 10 % SDS-PAGE, transferred to nitrocellulose membranes and incubated with a 1 : 1000 dilution of biotinylated *Sambucus nigra* (SNA) lectin (Vector Laboratories), followed by washing in PBS before addition of a 1 : 10 000 dilution of a horseradish peroxidase (HRP)–streptavidin conjugate (Sigma). The SuperSignal West Pico chemiluminescent ECL substrate (Thermo Scientific) allowed capture of signal on CL-XPosure film (Thermo Scientific) using a Tetenal developer and fixer in a Compact X4 Xograph imaging system.

#### Statistical analysis.

Comparisons of strains for biofilm growth and adhesion assays, and also the effects of enzyme inhibitors on biofilm growth and adhesion assays, were analysed by Student’s *t* test.

## Results

### NanH-dependent sialidase activity is important for biofilm growth using human glycoproteins as a source of sialic acid

To test whether *T. forsythia* can obtain sialic acid from relevant host glycoproteins for biofilm growth, 96-well plates were pre-coated with model glycoproteins at a similar concentration to that required for growth on free sialic acid and sialyl-lactose (6 mM) (Roy *et al.*, 2010). In parallel, filter-sterilized human whole saliva from healthy volunteers and human serum (purchased from Sigma) were used to mimic gingival crevicular fluid, as these represent typical secretions present in the oral cavity. Since *T. forsythia* sialidase activity can be inhibited by oseltamivir (Roy *et al.*, 2010), we tested the effect of this inhibitor on biofilm formation to examine whether any or all of the growth was reliant on sialic acid.

**Fig. 1.**
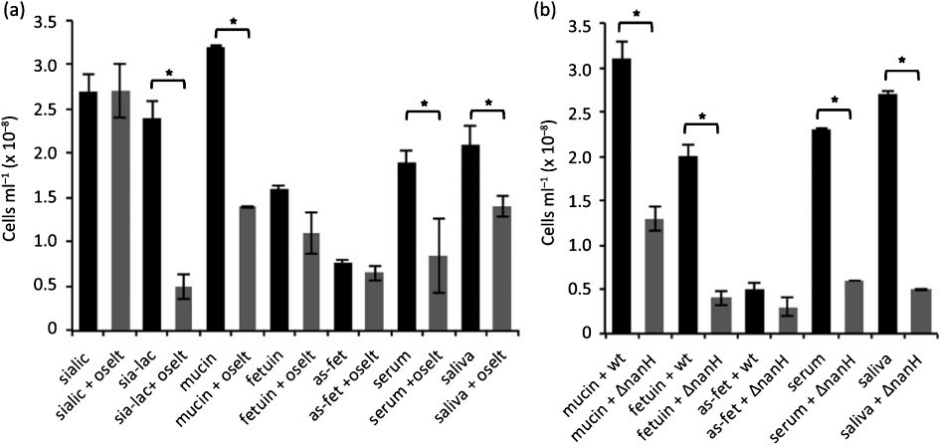
Growth of *T. forsythia* biofilm on glycoprotein surfaces. (a) Biofilm growth was assessed for the wild-type strain in the absence (black bars) or presence of oseltamivir (grey bars). Biofilms were set up in triplicate and wells were supplemented with 6 mM sialic acid (sialic) or 6 mM sialyl-lactose (sia-lac), or coated with 6 mM mucin, fetuin and asialofetuin (as-fet), 2 µg human serum ml^−1^ (serum) or saliva, with and without the addition of 10 mM oseltamivir to the TSB medium, as indicated at the time of inoculation. Glycoproteins were coated on the 96-well plate overnight at 4 °C and washed before inoculation with *T. forsythia* to a final OD_600_ of 0.05. Data are the mean number of cells with sds after harvesting from wells after 5 days. Differences between mean values in this experiment were evaluated by *t* test, with *P*<0.05 being taken as the level of significance (*statistically significant). (b) Biofilm growth was compared as above between the wild-type (wt) strain and the Δ*nanH* mutant strain of *T. forsythia* (ΔnanH).

Our previous data showed that biofilm growth on sialyl-lactose was inhibited fivefold by oseltamivir. We now highlighted the role of the NanH sialidase in this process, since the *nanH* deletion mutant was also unable to utilize sialyl-lactose for biofilm growth (Fig. 1). Similarly, biofilm growth on the heavily sialylated bovine submaxillary mucin was also reduced twofold by oseltamivir (*P*<0.0001) and the *nanH* deletion (*P*<0.005) (Fig. 1). While growth on fetuin was only marginally reduced by oseltamivir, the loss of NanH (in the Δ*nanH* mutant strain) caused a striking fourfold reduction (*P*<0.0005). In support of this observation, growth on asialofetuin compared with fetuin was much lower (*P*<0.02). In addition, biofilm growth on asialofetuin was higher (*P*<0.03) than the background when the cells were inoculated into media lacking NAM or sialic acid as a growth substrate, suggesting that the bacteria might use other sugar substrates present on asialofetuin. In order to test whether the sialidase activity had any influence on growth in a situation where there was a clinically relevant mixture of glycoproteins, we repeated the experiment on surfaces coated with human saliva and serum. Under these experimental conditions there were statistically significant two- to threefold reductions (in the presence of inhibitors) in growth (*P*<0.01) on human saliva and serum, suggesting that sialic acid might be an important *in vivo* growth substrate.

### NanH is required for efficient adhesion to sialoglycoproteins, saliva and serum

As is the case for many biofilm-forming bacteria, the initial adhesion to a surface is a key first step in the establishment of a stable community, such as the dental plaque (Kolenbrander & Palmer, 2004; Scannapieco, 1994). To test adhesion to sialoglycoproteins, saliva and serum bacteria were incubated with coated surfaces as for the biofilm growth experiments, but for just 3 h.

**Fig. 2.**
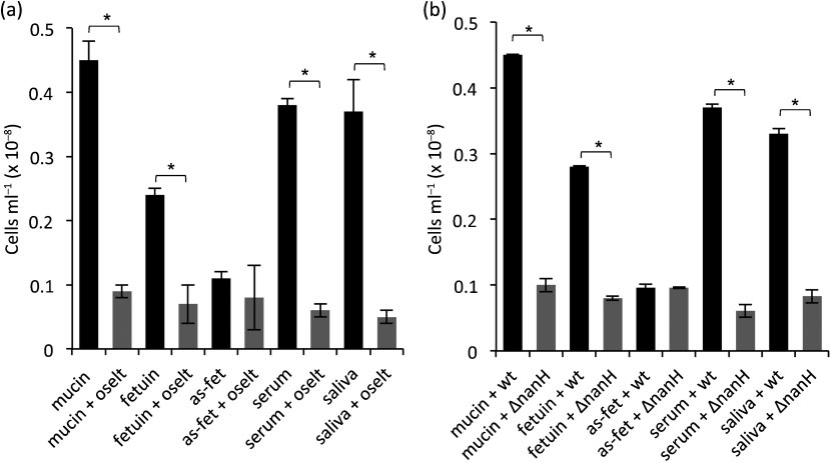
Initial attachment of *T. forsythia* to glycoprotein-coated surfaces. (a) Adhesion to glycoproteins was assessed for the wild-type strain in the absence (black bars) or presence of oseltamivir (grey bars). Biofilms were set up in triplicate and wells were supplemented with 6 mM sialic acid (sialic), or were coated with 6 mM mucin, fetuin and asialofetuin (as-fet), 2 µg human serum ml^−1^ (serum) or saliva, with and without the addition of 10 mM oseltamivir to the TSB medium, as indicated at the time of inoculation. Glycoproteins were coated on the 96-well plate overnight at 4 °C and washed before inoculation with *T. forsythia* to a final OD_600_ of 0.05. Data are the mean number of cells with sds after harvesting from wells after 3 h. Differences between mean values in this experiment were evaluated by *t* test, with *P*<0.05 being taken as the level of significance (*statistically significant). (b) Adhesion to glycoproteins was compared as above between the wild-type (wt) strain and Δ*nanH* mutant strain of *T. forsythia* (ΔnanH).

The inclusion of 10 mM oseltamivir in the medium resulted in reduced adherence of *T. forsythia* to the glycoproteins of between two- and fivefold (*P*<0.05) (Fig. 2a). demonstrating that the sialidase activity of *T. forsythia* is responsible for initial attachment to model sialylated glycoproteins (mucin and fetuin) and to the clinically relevant protein mixtures (serum and whole saliva). Again, adhesion to asialofetuin was lower than to the sialylated form, and no significant reduction was observed in the presence of the inhibitor. It is also worth noting that the variation in absolute levels of adhesion to different substrates may reflect differences in sialic acid content, but could also reflect the concentration of adhered protein, which is very difficult to estimate; nonetheless, the consistent reductions in adhesion in the presence of inhibitor are striking and indicate a role for sialidase in this event. When the adhesion assays were performed with the *nanH* sialidase mutant, it displayed a similar two- to fivefold reduction in attachment to the glycoproteins compared with the wild-type strain (Fig. 2b).

**Fig. 3.**
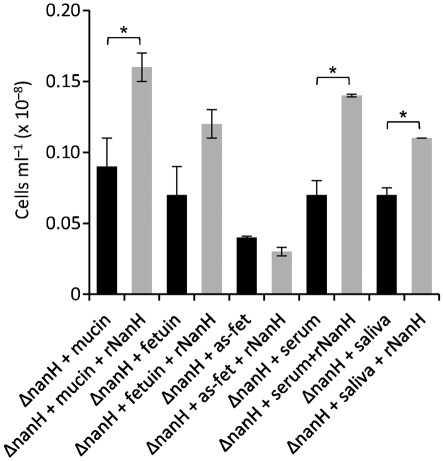
Adhesion of the Δ*nanH* mutant strain of *T. forsythia* to glycoprotein surfaces without (black bars) and with purified NanH (rNanH) (grey bars). Biofilms were set up in triplicate and wells were supplemented with 6 mM sialic acid (sialic) or 6 mM sialyl-lactose (sia-lac) to the TSB medium, or were coated with 6 mM mucin, fetuin and asialofetuin (as-fet), 2 µg human serum ml^−1^ (serum) or saliva, as indicated at the time of inoculation. Glycoproteins were coated on the 96-well plate overnight at 4 °C and washed before inoculation with *T. forsythia* to a final OD_600_ of 0.05. Data are the number of cells with sds after harvesting from wells after 3 h. Differences between mean values in this experiment were evaluated by *t* test, with *P*<0.05 being taken as the level of significance (*statistically significant).

Ideally, a genetic complementation experiment would be carried out in which the wild-type *nanH* gene was reintroduced into the *nanH* mutant strain. However, to date, no one has yet developed a genetic complementation system for *T. forsythia*. Therefore we used purified rNanH protein in an enzyme complementation study (Honma *et al.*, 2011). In these experiments the *nanH* deletion was incubated with glycoprotein-coated surfaces and cell attachment was assayed. Fig. 3 shows a statistically significant increase (*P*<0.05) in adhesion of between 1.5- and twofold in the presence of rNanH. This confirms that NanH is essential for initial attachment to the glycoproteins present in host cells or in the oral environment.

### NanH can directly release sialic acid from glycoproteins

**Fig. 4.**
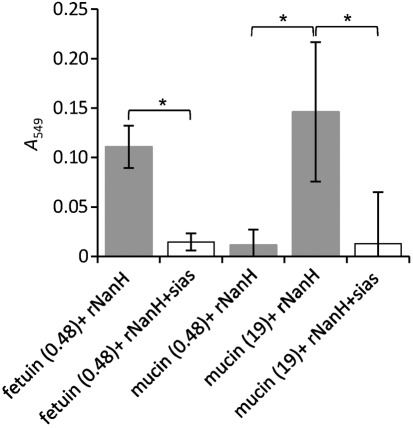
Release of free sialic acid from glycoprotein by recombinant NanH (rNanH). Six millimolar fetuin [0.48 µg sialic acid ml^−1^, fetuin (0.48)], 0.002 mM mucin [0.48 µg sialic acid ml^−1^, mucin (0.48)] or 6 mM mucin [19 µg sialic acid ml^−1^, mucin (19)] was incubated in the presence (white bars) and absence (grey bars) of 5 mM of the sialidase inhibitor siastatin B (+sias) at 37 °C for 3 h. *A*_549_ was measured after colour development (*statistically significant at *P*<0.05).

In order to more fully characterize the role of NanH in release of sialic acid from a range of glycoproteins, we purified rNanH as described previously (Honma *et al.*, 2011). After purification, the rNanH enzyme (0.78 mg ml^−1^) was incubated with 6 mM fetuin and mucin for 3 h at 37 °C. The release of sialic acid from these proteins was then assessed using the thiobarbiturate assay of Skoza & Mohos (1976). Fig. 4 illustrates that in addition to cleavage of the 4-methylumbelliferyl substrate, we have now shown, to our knowledge for the first time, that rNanH can cleave sialic acid from the sialylated glycoprotein fetuin (Fig. 4, column 1) with the release of 30 nM sialic acid min^−1^ (mg protein)^−1^. Furthermore, we showed that this is inhibitable by the sialidase inhibitor siastatin B, indicating that this is a sialic acid-specific activity. When this enzyme was incubated with the same concentration of mucin (6 mM bovine submaxillary) (Fig. 4, column 4) we also observed a release of sialic acid. This result was somewhat surprising, since Thompson *et al.* (2009) reported that they could not detect release of sialic acid from mucin using rNanH-expressing lysates of *E. coli*. However, the concentration of mucin used in this assay (6 mM) contained approximately 32-fold more sialic acid than the equivalent concentration of fetuin. In contrast, if rNanH was incubated with 0.002 mM mucin, which contained the equivalent amount of sialic acid (0.48 µg ml^−1^) as fetuin, there was almost no release of sialic acid (Fig. 4, column 3).

**Fig. 5.**
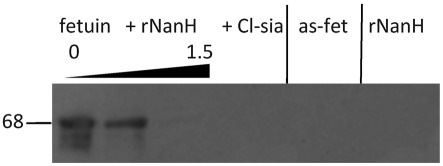
Removal of lectin-binding moieties by rNanH. Fetuin at 10 µg ml^−1^ was incubated with increasing concentrations of rNanH (0, 0.78 and 1.5 mg ml^−1^) for 3 h before running on an SDS-PAGE gel, blotting onto a nitrocellulose membrane, probing with 5 µg ml^−1^ biotinylated SNA lectin, and visualizing with streptavidin–HRP and luminescent substrate, before incubation with X-ray film. *Clostridium tetani* sialidase (Cl-sia) (50 U) was used as a positive control, with asialofetuin (as-fet) and 1.5 mg rNanH ml^−1^ as negative controls.

In order to further confirm our observation that rNanH can release sialic acid from physiologically relevant substrates, we probed the glycoprotein fetuin that had been pretreated with rNanH for the presence of the epitope for the sialic acid-specific biotinylated SNA lectin, which was detected with streptavidin–HRP. The SNA lectin binds preferentially to sialic acid linked to terminal galactose in α-2,6 and to a lesser extent α-2,3 linkages, both of which are contained in fetuin. Fig. 5 shows that in the absence of rNanH (lane 1), the fetuin band can clearly be seen at 68 kDa. However, after the treatment of fetuin with increasing concentrations of purified rNanH, the lectin-binding site was removed and the fetuin-specific band disappeared from the blots (Fig. 5). These data support our findings using the thiobarbituric acid assay, and demonstrate for the first time to our knowledge that *T. forsythia* NanH can cleave both α-2,6- and α-2,3-linked sialic acid directly from glycoproteins.

## Discussion

Our previous work identified that the fastidious anaerobe *T. forsythia* is able to utilize sialic acid as a growth factor to stimulate its biofilm growth, and that it possesses a novel sialic uptake transport system (Roy *et al.*, 2010). We have also shown that the sialidase activity of *T. forsythia* is important for sialic acid acquisition from the common glycosyl mimic sialyl-lactose (Roy *et al.*, 2010). In other work we have established that the NanH sialidase is involved in adhesion to and invasion into gingival epithelial cells (Honma *et al.*, 2011). We now report that the NanH sialidase is key to the initial formation and growth of biofilms on glycoprotein-coated surfaces, where the only source of sialic acid was conjugated to glycoproteins as α-2,3 or 2,6 linkages to galactose or glucose. This is particularly pertinent given the environmental niche that *T. forsythia* inhabits in the oral cavity, where there is an abundance of sialic acid-containing glycoproteins that include fibronectin, integrins, gangliosides and mucins, both in secretions and contained on the surface of oral epithelial cells (Gabriel *et al.*, 2005; Byres *et al.*, 2008; Martin *et al.*, 2005; Offner & Troxler, 2000; Dabelsteen, 1996). The role of sialidases in the release of nutritionally available sialic acid is similar to the strategy of several human-dwelling bacteria in mucin-rich environments, such as the intestinal (*Bacteroides fragilis* and *Vibrio cholerae*) and respiratory tracts (*S. pneumoniae*) (Corfield, 1992).

Our data highlight roles for conjugated sialic acid both as a source of nutrition and as a receptor in the initial stages of biofilm formation on glycoprotein-coated surfaces, a situation which may mimic *in vivo* conditions in which inert and cellular surfaces are coated in a layer of mucus (Derrien *et al.*, 2010). While binding of oral bacteria such as *Streptococcus gordonii* and *Streptococcus sanguis* to the sialic acid in mucin has been documented in the past (Demuth *et al.*, 1990; Takahashi *et al.*, 2002, 2004), we believe that this is the first indication that a Gram-negative periodontal pathogen also possesses such an activity. Our finding that both sialidase inhibitors and deletion of the sialidase gene affect adhesion to glycoproteins suggests that three separate mechanisms may be occurring simultaneously to achieve adhesion: (1) the sialidase itself acts as a lectin-like adhesin (Banerjee *et al.*, 2010), perhaps via the 170 aa N-terminal region; (2) the sialidase causes exposure of the underlying glucose and galactose residues to which the bacteria bind; (3) there is a two-stage mechanism in which sialic acid is key to the initial stages of adhesion but in which underlying sugars may be involved in more stable interactions. This is the case for some other pathogens, in which desialylated glycolipids can act as receptors for pathogenic bacteria, including *S. pneumoniae* and *Pseudomonas aeruginosa*, which bind to exposed GalNAcβ1-4Gal residues when sialic acid is removed (Krivan *et al.*, 1988). Taken overall and in light of our previous work showing the importance of sialidase and sialic acid in adhesion to host cells, it seems that *in vivo* desialylation of host molecules is crucial to *T. forsythia.* Recent evidence from the Fletcher laboratory has highlighted that this may also be the case for the other major periodontal pathogen *P. gingivalis*, in which mutations in a putative sialidase and two sialyl-endopeptidases affect adhesion to HeLa cells (Aruni *et al.*, 2011); however, at present there is no evidence regarding their role in biofilm formation in *P. gingivalis*. Similarly, in the oral setting, the sialidase activity of several *Streptococcus* spp. can also result in exposure of galactose residues, enhancing the attachment of bacteria that possess galactosyl-binding adhesins, e.g *Fusobacterium* spp. (Falkler & Burger, 1981) and *Actinomyces viscosus* (Ellen *et al.*, 1980). It now seems possible that *T. forsythia* may also play such a role.

While the role of sialidase in *T. forsythia* adhesion and nutrition seems well established, like many members of the Bacteroidetes, *T. forsythia* possesses a plethora of glycosidase and sugar-focussed metabolic capabilities. For example *T. forsythia* possesses at least three putative beta-hexosaminidase-encoding genes (*TF0036*, *TF2925* and *TF0014*), which may possess the ability to remove terminal galactose and glucose sugars from glycoproteins that are exposed after removal of sialic acid (Spiro & Bhoyroo, 1974; Salyers *et al.*, 1988). However, at present, the role of these enzymes in the biofilm lifestyle of *T. forsythia* is unknown.

The action of sialidase may also have nutritional consequences for other oral biofilm inhabitants; for example, *S. oralis* releases sialic acid from α_1_-acid glycoproteins (AGPs), which are then utilized by other bacteria (Byers *et al.*, 1999). Similarly, after the removal of sialic acid from fetuin and mucin, subsequent removal of galactose and glucose can be used by bacteria as a carbon and nitrogen source (Leake & Read, 1990). Indeed, this may be the case for *T. forsythia*, since subgingival plaque is made up of several species with the ability to use sialic acid and other sugars, while *T. forsythia* itself probably cannot.

In addition to our experiments highlighting adhesion to and biofilm growth upon glycoprotein-coated surfaces, we also showed by biochemical and lectin-binding assays that the rNanH enzyme releases α-2,6-linked sialic acid from fetuin. This was expected, given the fact that NanH has been shown to preferentially cleave sialic acid from 2,6 sialyl-lactose *in vitro.* Our finding that sialic acid release from the α-2,3- and α-2,6-linked but largely 9-*O*-acetylated sialic acid contained in mucin was much less efficient is also in keeping with earlier findings, since this chemical group is known to inhibit bacterial sialidases (Thompson *et al.*, 2009).

However, our data show that *T. forsythia* is able to grow as a biofilm on mucin-coated surfaces and indicate that it is thus able to remove the 9-*O*-acetyl group from the sialic acid contained on the surface of mucin (Fig. 4). This is probably due to the activity of a putative secreted 9-*O*-acetylesterase encoded by the *TF0037* gene, and may play a crucial role *in vivo* in the mucin-rich oral environment that *T. forsythia* inhabits. In the gut bacterium *E. coli*, mutation of a recently identified sialate-*O*-acetyl esterase, *nanS*, results in abolition of its ability to use 9-*O*-acetylated sialic acid as a sole carbon and energy source. However, the NanS of *E. coli*, which does not possess a secreted sialidase, is a predicted periplasmic protein that probably acts to process Neu5,9Ac during sialic acid uptake (Steenbergen *et al.*, 2009). In contrast, the putative NanS of *T. forsythia* is likely to be secreted into the extracellular milieu, where it could help with scavenging of sialic acid from host sources.

Overall, our findings indicate that sialoglycoproteins are important substrates for biofilm formation and attachment of *T. forsythia* to oral surfaces *in vivo*. In addition, this study raises the possibility that oseltamivir or siastatin B might be used as alternative drug therapies for reduction of dental plaque biofilms.
